# Association of female sex with cataract surgery in the general population but not in plaque brachytherapy-treated uveal melanoma patients

**DOI:** 10.1038/s41598-024-73346-3

**Published:** 2024-09-24

**Authors:** Anna Hagström, Shiva Sabazade, Viktor Gill, Gustav Stålhammar

**Affiliations:** 1 Department of Clinical Neuroscience, Division of Eye and Vision, St. Erik Eye Hospital, Karolinska Institutet, Eugeniavägen 12, 17164 Stockholm, Sweden; 2 Ocular Oncology Service, St. Erik Eye Hospital, Stockholm, Sweden; 3 Department of Pathology, Västmanland Hospital Västerås, Västerås, Sweden; 4 St. Erik Ophthalmic Pathology Laboratory, St. Erik Eye Hospital, Stockholm, Sweden

**Keywords:** Cataract, Sex, Incidence, Risk factors, Sex, Age, Brachytherapy, Eye cancer, Cancer, Oncology

## Abstract

Cataract is a leading cause of blindness worldwide, necessitating a deeper understanding of its risk factors. We analyzed two cohorts: 1000 individuals from the general Swedish population and 933 patients who received plaque brachytherapy for uveal melanoma. Using Kaplan-Meier and cumulative incidence analyses, as well as Cox and competing risk regressions, we assessed whether there is a relationship between sex and cataract surgery. In the general population, female sex was a significant risk factor for cataract surgery, with a 10-year incidence of 16% compared to 10% for males (subdistribution hazard ratio adjusted for age, 1.35, *P* < 0.001). In the brachytherapy cohort, female sex was not associated with an increased incidence of cataract surgery, with a 10-year incidence of 25% versus 23% for males (HR 1.08, *P* = 0.61). Visual acuity at the time of cataract surgery did not significantly differ between sexes in either cohort, suggesting that differences in surgery rates are not due to health-seeking behavior or surgery assessment thresholds. These findings indicate that female sex is associated with a higher risk of cataract surgery in the general population, but not among those treated with plaque brachytherapy for uveal melanoma.

**Supplementary Information:**

The online version contains supplementary material available at 10.1038/s41598-024-73346-3.

## Introduction

Cataract is the leading cause of blindness worldwide, presenting a significant public health challenge^1,2^. In Sweden alone, cataract surgery is performed on over 140 000 individuals annually, representing about 1.4% of the population^3^. The high prevalence of cataract and its subsequent surgery underscores the importance of understanding its risk factors, which include increasing age, diabetes, intravitreal injections, and female sex^4–6^.

In most societies, women tend to outlive men, which may contribute to a higher incidence of cataracts among women^7^. However, even after adjusting for age in multivariate models, female sex has emerged as an independent risk factor for cataract^8^. This raises the question: Is female sex truly associated with an increased risk for cataract, or is this association confounded by the competing risk of death? In the context of time-to-event analyses, a competing risk is an event that precludes the occurrence of the primary event of interest. For instance, in a study on the onset of cataract as the primary event, death emerges as a competing risk by preventing the potential development of cataract. Traditional survival models, such as Kaplan-Meier analyses and Cox regressions, censor patients at the time of death. This approach may inadvertently bias the analysis by removing older men—who are more likely to die than women of the same age and who are more likely to develop cataracts than younger men—thereby potentially skewing the observed association between sex and cataract risk.

In the landmark Beaver Dam Eye Study it was observed that women who accepted the invitation for an examination had a higher likelihood of developing nuclear cataract compared to men, even after adjustments for age were made and accounting for the competing risk of death^9^. This reinforces the association between female sex and a greater incidence of nuclear cataract. However, the decision to stratify age into four strata, although it aids in simplifying the analysis and its interpretation, might lead to an unintentional loss of critical information. Important differences in risk within each age group could be obscured, as this method assumes homogeneity of risk within each stratum.

Additionally, age-adjusted analysis from another segment of the Beaver Dam Eye Study revealed that men were more likely than women to experience a significant worsening of vision—specifically, a doubling of the visual angle (indicative of a decline of 15 letters or more in visual acuity in the better eye) for any reason, including cataract, at the 15-year follow-up. This offers important insights into the dynamics of sex, visual acuity, and cataract surgery rates^10^. Despite previous findings that visual acuity between men and women at the time of cataract surgery is comparable, with one study noting better visual acuity for women only in the 55–64 years age group, this discrepancy suggests potential behavioural differences influencing cataract surgery rates^11^. This could be attributed to variations in patient-initiated healthcare seeking or in how healthcare providers assess the indications for surgery.

To adequately address the competing risk, our study will employ a formal competing risk regression analysis. We aim to assess whether there is a relationship between sex and cataract surgery in a sample of 1000 individuals from the general Swedish population, using competing risk incidence data and multivariate competing risk regressions. Additionally, we will include 933 patients who underwent plaque brachytherapy for uveal melanoma, an aggressive primary intraocular malignancy, to determine whether the sex-based risk for cataract persists in the context of radiation exposure^12,13^. We will also examine visual acuity at cataract surgery admission to gauge if differences in healthcare-seeking behaviours or healthcare provider assessments contribute to disparities in surgery rates between genders. By addressing the limitations of traditional survival analysis and drawing from a diverse patient sample, our study aims to provide a more nuanced understanding of the relationship between sex and cataract surgery incidence.

## Results

### Descriptive statistics

Among the 1933 included individuals, 930 were female (48%). The distribution of sex was similar between the 1000 individuals in the general population sample and the 933 patients treated with plaque brachytherapy (Holm-Bonferroni corrected χ^2^* P* > 0.99). No individuals in the general population sample were diagnosed with uveal melanoma or treated with plaque brachytherapy during the follow-up period. When pooling the general population and brachytherapy samples, there was no significant difference in age on January 1st, 2010, between males and females (median age 62 and 61 years, respectively; interquartile range [IQR] 18 and 20 years, respectively; Mann-Whitney *U P* = 0.43). Additional details regarding the general population and brachytherapy samples are provided in Table 1.

**Table 1:** Patient and sample characteristics.

	General population sample, n = 1000	Brachytherapy sample, n = 933	P*
Sex, n(%)			> 0.99**
Female	484 (48)	446 (48)	
Male	516 (52)	487 (52)	
Age on January 1st, 2010, median years (IQR)			
Males	64 (15)	60 (18)	> 0.99^†^
Females	61 (22)	60 (17)	> 0.99^†^
Age at uveal melanoma diagnosis, median years (IQR)			> 0.99^†^
Males		65 (17)	
Females		66 (18)	
BCVA at uveal melanoma diagnosis, mean LogMAR (SD)		0.68 (0.59)	
BCVA at cataract surgery, mean LogMAR (SD)	0.44 (0.31)	0.90 (0.65)	0.003^†^
Cataract surgery^‡^, n			
Males	84	128	
Females	103	130	
Secondary enucleation, n			
Males	0	13	
Females	0	14	
All-cause mortality, n			
Males	68	104	
Females	74	89	

BCVA, best corrected visual acuity. IQR, interquartile range. SD, standard deviation. *Holm-Bonferroni corrected values. **Chi-square test. ^†^Mann-Whitney *U* test. ^‡^Of which 86 patients in the brachytherapy sample had undergone cataract surgery prior to plaque brachytherapy.

A cross-reference of the 933 uveal melanoma patients and the 1000 individuals from the general population, with records from 1 515 983 cataract surgeries between 2010 and 2022, revealed that 84 males and 103 females from the general population sample, and 128 males and 130 females from the brachytherapy sample had undergone cataract surgery by the data collection endpoint. In the brachytherapy sample, no significant differences were observed between males and females in terms of tumor size, tumor location, type of radioisotope used, apical dose, or scleral dose (Supplementary Table 1). Eighty-six patients had undergone cataract surgery prior to plaque brachytherapy and were excluded from further outcome analyses, including Kaplan-Meier, Cox regressions, competing risk incidence analyses, and visual acuity at the time of cataract surgery. Patients in the brachytherapy sample had significantly worse BCVA when being admitted for cataract surgery (Holm-Bonferroni corrected Mann-Whitney *U P* < 0.001). Mortality, irrespective of cause, was recorded for 68 males and 74 females from the general population sample, and 104 males and 89 females from the brachytherapy sample. Additionally, post-primary plaque brachytherapy, 27 uveal melanoma patients underwent secondary enucleation. Our analysis confirmed that the data on time-to-cataract surgery adhered to the proportional hazards assumption. This was supported by the examination of log-minus-log-transformed survival curves, showing neither crossing nor divergence, and the global test based on Schoenfeld residuals, which indicated no significant evidence of time-varying effects (*P* = 0.85, Supplementary Fig. 1). The median times to cataract surgery, as well as to secondary enucleation (for the brachytherapy sample only), death from any cause, or last follow-up, have been detailed in our previously published work^14^.

### Kaplan-Meier analyses

**Fig. 1:**
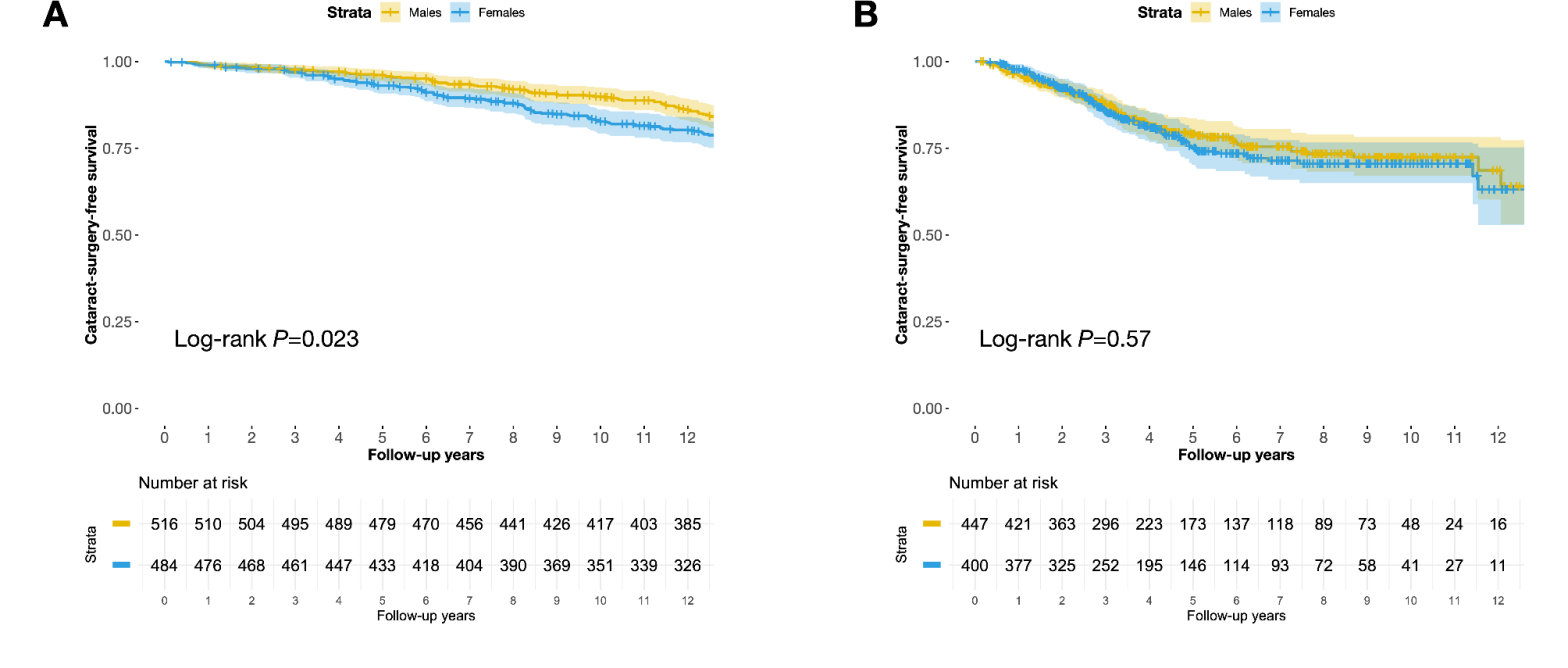
Kaplan-Meier curves for cataract-surgery-free survival. (**A**) General Population Sample; (**B**) Brachytherapy Sample, excluding 86 patients who had undergone cataract surgery prior to receiving plaque brachytherapy.

Within the general population sample, female individuals had worse cataract-surgery-free survival compared to males. Specifically, the proportions of cataract-surgery-free survival at 5, 10, and 12 years for females were 93% (95% CI 0.91–0.95), 83% (95% CI 0.79–0.86), and 80% (95% CI 0.77–0.84), respectively; while for males, these were 96% (95% CI 0.94–0.98), 90% (95% CI 0.87–0.93), and 86% (95% CI 0.83–0.89), respectively (Log-rank *P* = 0.023, Fig. 1A). Conversely, in the brachytherapy sample, the survival differences between sexes were not statistically significant: The cataract-surgery-free survival rates for females at 5, 10, and 12 years were 75% (95% CI 0.70–0.80), 71% (95% CI 0.65–0.77), and 63% (95% CI 0.53–0.75), respectively; compared to 79% (95% CI 0.75–0.84), 72% (95% CI 0.67–0.78), and 69% (95% CI 0.60–0.78) for males (Log-rank *P* = 0.57, Fig. 1B).

### Cox regression and competing risk analyses

**Fig. 2:**
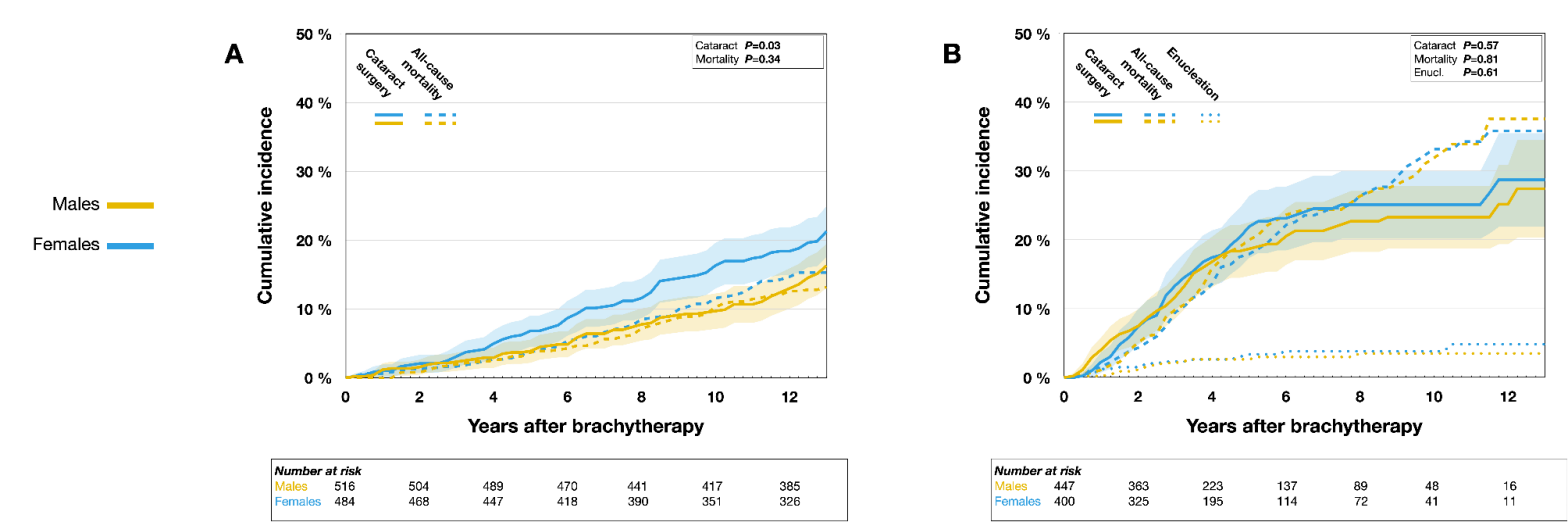
Cumulative incidence of cataract surgery from competing risk data. (**A**) General Population Sample; (**B**) Brachytherapy Sample, excluding 86 patients who had undergone cataract surgery prior to receiving plaque brachytherapy.

Within the general population sample, females demonstrated a higher incidence of cataract surgery when compared to males, with cumulative incidences at 5, 10, and 12 years of 0.07 (95% CI 0.05 to 0.09), 0.16 (95% CI 0.13 to 0.20), and 0.18 (95% CI 0.15 to 0.22) for females; and 0.04 (95% CI 0.02 to 0.06), 0.10 (95% CI 0.07 to 0.12), and 0.13 (95% CI 0.10 to 0.16) for males at the same intervals (Gray’s test *P* = 0.03, Fig. 2A). In the brachytherapy sample, the incidences for females at 5, 10, and 12 years were 0.22 (95% CI 0.17 to 0.26), 0.25 (95% CI 0.20 to 0.30), and 0.29 (95% CI 0.22 to 0.36); and for males, 0.19 (95% CI 0.15 to 0.23), 0.23 (95% CI 0.19 to 0.28), and 0.25 (95% CI 0.19 to 0.31), respectively, with no significant difference observed (Gray’s test *P* = 0.57, Fig. 2B).

In multivariate Cox regressions adjusted for patient age, female sex was associated with an increased likelihood of cataract surgery in the general population sample (HR 1.42, Holm-Bonferroni corrected *P* = 0.04), whereas no significant association was found in the brachytherapy sample (HR 1.08, Holm-Bonferroni corrected *P* = 0.63, Supplementary Table 2).

Similarly, multivariate competing risk regressions with patient age as a covariate revealed that female sex was associated with cataract surgery in the general population sample, but again, not in the brachytherapy sample (Table 2)**.**

**Table 2:** Multivariate competing risks regressions, subdistribution hazard ratio exp(β_j_) for cataract surgery.

		β_j_	S.E.	z	P*	exp(β_j_)	95% CI	Wald
General population sample, n =1000	Multivariate							
	Sex^a^	0.30	0.15	2.0	< 0.001	1.35	1.01 to 1.80	4.2
	Age^b^	0.03	0.01	6.6	< 0.001	1.03	1.02 to 1.04	43.3
Brachytherapy sample, n = 847	Multivariate							
	Sex^a^	0.08	0.15	0.5	0.61	1.08	0.80 to 1.46	0.3
	Age^b^	0.02	0.01	3.0	0.005	1.02	1.01 to 1.03	9.1

^a^Female versus male (categorical variable). ^b^Per increasing year (continuous variable). *Holm-Bonferroni corrected value. S.E., standard error. *z*, Z-score. For the general population sample, all-cause mortality was a competing risk to cataract surgery. For the brachytherapy sample, both all-cause mortality and enucleation were competing risks.

### Visual acuity at the time of cataract surgery

**Fig. 3:**
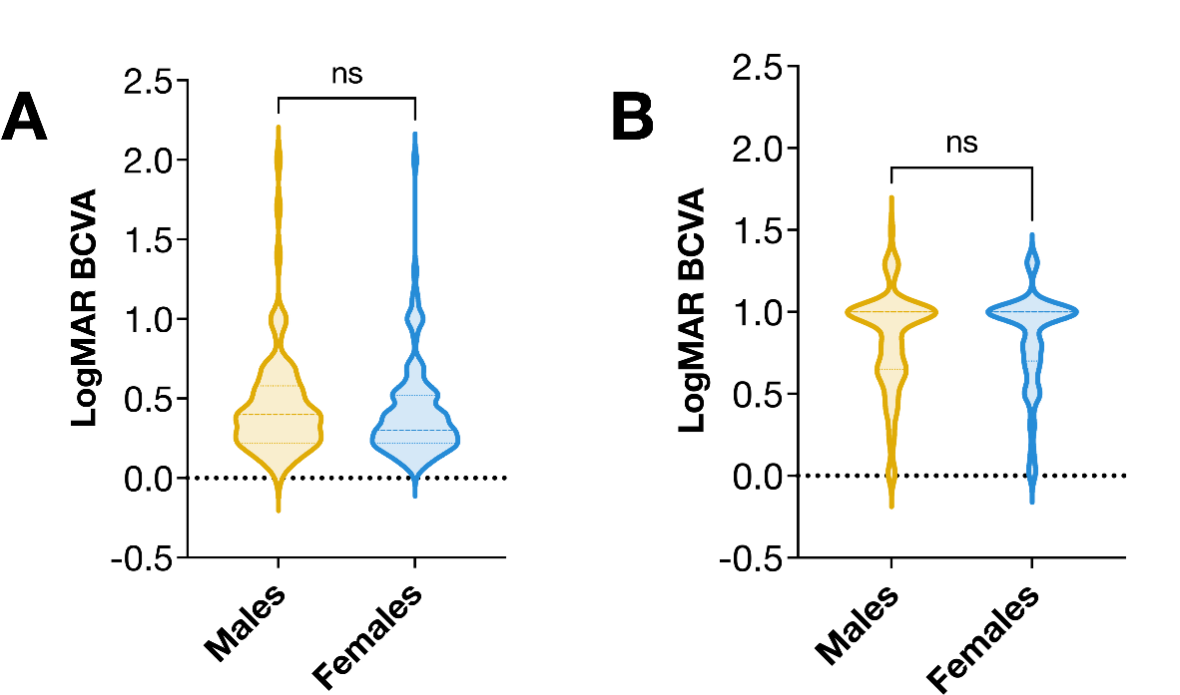
Best corrected visual acuities (BCVA) at the time of admission for cataract surgery for patients in (**A**) the general population sample, and (**B**) the brachytherapy sample. No significant differences were observed between males and females according to the Mann-Whitney U test. ns, non-significant.

At the time of admission for cataract surgery, the median BCVA was LogMAR 0.3 (IQR 0.3) for females and LogMAR 0.4 (IQR 0.4) for males in the general population sample (Holm-Bonferroni corrected Mann-Whitney *U P* = 0.62, Fig. 3A). In the brachytherapy sample, both females and males had a median BCVA of LogMAR 1.0 (IQR 0.3; Holm-Bonferroni corrected Mann-Whitney *U P* = 0.75, Fig. 3B). These findings indicate that despite the significantly worse visual acuity observed in the brachytherapy sample overall, there were no significant differences in visual acuity between sexes within either the general population or the brachytherapy sample. Therefore, it is unlikely that differences in health-seeking behavior or thresholds for admission to surgery explain the observed difference in cataract surgery incidence between the sexes. If females had been more likely to seek medical attention or if there had been a lower threshold for recommending surgery to females, we would expect to see a greater disparity in visual acuity between sexes at the time of surgery. However, since visual acuity was similar between females and males, these factors are unlikely to account for the difference in cataract surgery incidence.

## Discussion

### Key findings

In this study, we demonstrate that female sex is a risk factor for cataract surgery in a general population sample, but not among patients treated with plaque brachytherapy for uveal melanoma. This tendency was consistent in Kaplan-Meier and cumulative incidence analyses, and in Cox- and competing risk regressions. The similarity of visual acuity levels for men and women at the time of admission for cataract surgery makes differences in health-seeking behaviour or assessment thresholds for surgery an unlikely reason for the differences in cataract surgery rates.

## Possible causes

Several previous studies have observed a higher incidence of cataract surgery as well as disability from cataract among females in the general population^9,15–17^. Mortality among uveal melanoma patients, on the other hand, does not seem to differ significantly between the sexes^18,19^. One study noted a lower incidence of metastatic death in females during the first decade following brachytherapy but a higher incidence in later years^20^. Oxidative stress has been implicated as a central pathogenic mechanism in the formation of cataracts. This is supported by a wealth of epidemiological data indicating that UVB irradiation and smoking are significant risk factors. Additionally, evidence from animal and cell culture models suggests that oxidative stress can induce lens opacification akin to the changes observed in the aging human lens^21,22^.

The absence of a sex-based difference in cataract surgery rates among patients treated with plaque brachytherapy for uveal melanoma is intriguing and warrants further investigation. In previous studies using rodent models to explore radiation-induced cataract, the presence of ovaries was associated with an increase in lens opacifications, and the administration of E2 to ovariectomized animals was found to accelerate the progression of cataract^23^. Conversely, transgenic mice expressing a dominant-negative form of the estrogen receptor α (ERα), which inhibits ERα function, displayed spontaneous development of cortical cataract in females post-puberty^24^. Ovariectomy performed prior to sexual maturation, but not after, was effective in preventing lens opacification, indicating a requisite role for estrogens in activating the ERΔ3 repressor. This notion was bolstered by findings that exogenous estrogen induced cataract formation in ERΔ3 transgenic mice, affecting both males and females, thus suggesting the importance of estrogen and its receptors in maintaining lens homeostasis across both sexes. Consequently, estrogen appears to offer a protective effect against cataractogenesis induced by various insults. However, the timing of estrogen exposure is crucial, and there may also be receptor-independent mechanisms through which estrogen acts as a cataractogenic factor. Physiological concentrations of 17β-estradiol (E2) have been shown to confer protection against oxidative stress in cultured lens epithelial cells, leading to the hypothesis that the increased risk of cataract in women could be attributed to the decrease in estrogen levels at menopause^25^.

Given the significantly elevated incidence of cataract surgery following plaque brachytherapy, it is plausible to speculate that the relatively limited protective effect of male sex is overshadowed by the strong cataractogenic impact of radiation^14,26,27^. This supposition is in line with extensive research suggesting that, although estrogen might confer protection against cataract under specific circumstances, the profound radiation exposure inherent to brachytherapy might override these sex-related protective effects. This underscores the intricate dynamics among biological sex, hormonal influences, and environmental or therapeutic exposures in the development of cataracts. Additionally, the routine ophthalmological follow-ups for brachytherapy patients could lead to a differential detection and treatment pattern for cataracts compared to the general populace, potentially affecting observed surgery rates.

## Limitations

This study is based on two retrospective cohorts, which may introduce biases related to patient selection and data accuracy. The inherent nature of retrospective non-randomized analysis limits our ability to control for unknown confounders that could influence cataract surgery rates.

Additionally, employing age as a continuous variable in competing risk regressions, while allowing for a detailed analysis, complicates the interpretation of its effects. This is particularly true if the relationship between age and the risk of cataract surgery or death is not linear. Our analysis did not employ advanced modeling techniques, such as spline functions, that might better capture these non-linear effects.

A further limitation arises from the competing risk regression model’s assumption that the effects of covariates on the hazard rate of an event are constant over time. This model assumes a consistent impact of covariates, which may not hold true across different time periods, potentially leading to biased estimates. Furthermore, variables such as tumor thickness, location, and apical dose were not included in the regression analyses. Although these factors could potentially add value, including them would reduce statistical power, particularly given that no significant differences were observed between males and females for these variables. Consequently, we opted not to incorporate them into the regression models.

Lastly, while our study highlights associations between sex and cataract surgery incidence, it cannot establish causative mechanisms. The observational design precludes definitive conclusions about causality. Prospective studies, particularly those examining patients treated with plaque brachytherapy, are crucial. Such studies should meticulously document lens opacities and consider the radiation dose to the lens, to ascertain the true extent of opacities and any sex-related differences in their development.

## Conclusions

Our study demonstrates that female sex is associated with an increased risk of undergoing cataract surgery in the general population, a pattern that does not hold for patients treated with plaque brachytherapy for uveal melanoma. Additionally, there is no difference in visual acuity between males and females at the time of cataract surgery admission across both cohorts, suggesting that the observed gender differences in surgery rates cannot be attributed to disparities in clinical assessments or decision-making regarding the timing of the intervention. These findings highlight the influence of biological sex on cataract surgery incidence in the general population and point to the need for in-depth research to explore how specific treatments for conditions like uveal melanoma may alter traditional risk factors for cataract development.

## Methods

### Patients and samples

This study relies on two patient cohorts: the first comprising 1000 individuals from the general Swedish population (general population sample) and the second of 933 patients diagnosed with uveal melanoma, who underwent plaque brachytherapy treatment (brachytherapy sample). These cohorts have been previously published^14^. The brachytherapy sample encompasses all 933 patients receiving treatment for tumours affecting the choroid and/or ciliary body using ruthenium-106 or iodine-125 plaque brachytherapy at St. Erik Eye Hospital in Stockholm, Sweden, from January 1, 2010, through December 31, 2022. Patients diagnosed with iris melanoma were not included in this sample.

During this period, St. Erik Eye Hospital remained the sole institution in Sweden authorized to treat uveal melanoma with plaque brachytherapy, ensuring comprehensive coverage of all patients. Ruthenium plaques and iodine seeds were supplied by Eckert & Ziegler BEBIG, Berlin, Germany. The source specification data provided by the manufacturer were independently verified by medical physicists at Karolinska University Hospital, Stockholm. All primary tumours were diagnosed by ocular oncologists using slit-lamp biomicroscopy, indirect ophthalmoscopy, A- and B-scan ultrasonography, fundus imaging, and optical coherence tomography (OCT) as required. Transvitreal biopsies were performed if a diagnosis could not be established from the clinical examination alone.

Plaque brachytherapy with ruthenium-106 was typically reserved for tumours with an apical thickness of less than 6 mm, while thicker tumours were treated with iodine-125. For tumors ≤ 11 or ≤ 16 mm in largest basal diameter (LBD), CCA or CCB plaques were used, respectively, as as previously described^28^. Tumors with an apical thickness greater than 10 mm or a diameter exceeding 16 mm were typically managed with enucleation. Extrascleral extension was also an indication for enucleation, unless minimal. Proximity of the tumour to the optic disc (e.g., less than 2 mm) was not considered an absolute contraindication for brachytherapy, as previously reported^29^. The brachytherapy procedure was performed under general anaesthesia.

Clinicopathological and follow-up data, including secondary enucleation and mortality, were collected from our treatment register. The study adhered to the tenets of the Declaration of Helsinki, and approval was obtained from the Swedish Ethical Review Authority (reference 2022-00930-02). The requirement for informed consent from the study subjects was waived by the Swedish Ethical Review Authority on the condition that biological tissues from living patients were not included. Further, this retrospective chart review did not influence patient management (i.e., treatment, testing, follow-up, or information to patients), and was based on already collected data. The Strengthening the Reporting of Observational Studies in Epidemiology (STROBE) guidelines for reporting observational studies were used (available as a supplementary file).

The general population sample comprises 1000 individuals alive as of January 1, 2010. This sampling was derived from the population register, integrated into our digitized medical record system. To facilitate a balanced comparison, this cohort was matched to the brachytherapy sample based on sex and birth year, with specific attention to the following criteria:

- **Sex**: The distribution by sex in the general population sample was aligned with that of the brachytherapy sample, maintaining a deviation of no more than ± 2% points.
- **Birth Year**: The selection of individuals from the general population for each birth year was calibrated to reflect the composition of the melanoma cohort, permitting a variance of up to 5 individuals. For instance, if the melanoma group included 20 patients born in 1955, our selection from the general population would range from 15 to 25 individuals born in the same year, who were still alive in 2010.

Through cross-referencing with the National Cataract Register, we were able to ascertain which participants from this cohort had undergone cataract surgery. Importantly, this process did not require opening medical records or collecting any personal identifiers, such as personal identity numbers, names, addresses, contact information, or photographs.

### Swedish National Cataract Register

The general population and brachytherapy samples were cross-checked with data from the Swedish National Cataract Register (NCR) for the years 2010 through 2022. The NCR has been estimated to capture 93% of all cataract surgeries in the country. The variables in the core register for all cataract procedures have been published, and their changes over the years since the foundation in 1992 are documented in the Swedish meta-database Register Utilization Tool through the Swedish Research Council^3^. For all matches in the NCR, we collected data on the date of cataract surgery (of the tumour eye for uveal melanoma patients, of the first eye undergoing cataract surgery for individuals from the general population).

### Best corrected visual acuity (BCVA)

Each patient’s visual acuity (VA) was measured using a standardized methodology previously described^30,31^. Briefly, at the time of admission for cataract surgery, BCVA was assessed by an optometrist, ophthalmic nurse, or ophthalmologist. The assessment was conducted using a KM-chart in an illuminated light box, with patients tested at a distance of 3 m. The BCVA recorded was the smallest line on which five out of five, or six out of seven letters, were correctly identified after subjective refraction and correction in a trial frame. Patients were permitted to wear their own spectacles if appropriate. The method for BCVA measurement remained consistent throughout the study period and was not modified based on whether patients had been treated with plaque brachytherapy.

### Statistical methods

IBM SPSS Statistics version 29 (Armonk, NY, USA) and R version 4.2.2 (R Core Team, Vienna, Austria), with the survival, survminer, cmprsk, and ggplot2 packages, were used for statistical analyses. Kaplan-Meier curves for cataract surgery-free survival were plotted for males versus females in both the general population and brachytherapy samples. Similarly, cumulative incidence function estimates from competing risk data were plotted, with curves for both the incidence of cataract surgery and all-cause mortality^32^. The equality of survival distributions was tested with Gray’s test for equality. Cox- and competing risk regressions for the association with cataract surgery were calculated, with patient sex (categorical variable, male or female) and age at baseline (year 2010 for individuals from the general population, day of diagnosis for uveal melanoma patients, continuous variable e.g., 62 years) as covariates^33^. To determine whether our follow-up data met the proportional hazards assumption, we used a graphical approach and assessed log-minus-log-transformed survival curves and Schoenfeld residuals for time to cataract surgery. If *P* was > 0.05 in the Schoenfeld global test of proportional hazards, and if survival curves were parallel without any crossing or divergence, the proportional hazards assumption was considered fulfilled. Differences were considered statistically significant at *P* < 0.05, with all tests being two-sided to assess effects in both directions. The Holm-Bonferroni correction method was applied to all multiple comparisons to control for the false discovery rate.

## Electronic supplementary material

Below is the link to the electronic supplementary material.

Supplementary Material 1

## Data Availability

The data that support the findings of this study are available from Registercentrum Syd (https://rcsyd.se) but restrictions apply to the availability of these data, which were used under license for the current study, and so are not publicly available. Data are however available from the corresponding authors upon reasonable request and with permission of Registercentrum Syd and the Swedish Ethical Review Authority.
