# Supplementary material for: Association of female sex with cataract surgery in the general population but not in plaque brachytherapy-treated uveal melanoma patients
Source: Sci Rep. 2024 Sep 24;14:22016. doi: 10.1038/s41598-024-73346-3 (PMC11422500; doi:10.1038/s41598-024-73346-3)
Supplement: Supplementary file 1 — Supplementary Material 1 [file 41598_2024_73346_MOESM1_ESM.pdf]

Supplementary material

Female Sex is Associated with Higher Risk for Cataract in the General Population but Not in Patients Treated with Plaque Brachytherapy for Uveal Melanoma

Anna Hagström[1], Shiva Sabazade[1,2], Viktor Gill[1,3], Gustav Stålhammar[1,2,4]

[1]Department of Clinical Neuroscience, Division of Eye and Vision, Karolinska Institutet, Stockholm, Sweden
[2]Ocular Oncology Service, St. Erik Eye Hospital, Stockholm, Sweden
[3]Department of Pathology, Västmanland Hospital Västerås, Västerås, Sweden
[4]St. Erik Ophthalmic Pathology Laboratory, St. Erik Eye Hospital, Stockholm, Sweden

Table of contents

SUPPLEMENTARY TABLE 1 2
SUPPLEMENTARY FIGURE 1 3
SUPPLEMENTARY TABLE 2 4

Supplementary Table 1. Tumor and Treatment Characteristics in the Brachytherapy Cohort

	Males (*n*=487)	Females (*n*=446)	*P**
**Tumor apical thickness, mean mm (SD)**	5.1 (2.6)	5.2 (2.6)	0.95†
**Tumor LBD, mean mm (SD)**	10.5 (3.6)	10.8 (3.4)	0.57†
**Tumor location, *n* (%)‡**			>0.99**
Anterior to equator	24 (5)	19 (4)	
Posterior to equator	278 (57)	263 (59)	
Posterior pole	35 (7)	28 (6)	
N/a	150 (31)	136 (30)	
**Radioisotope, *n* (%)**			0.48**
Ruthenium-106	345 (71)	294 (66)	
Iodine-125	142 (29)	152 (34)	
**Apical dose, mean Gy (SD)**	94 (12)	93 (13)	0.41†
**Scleral dose, mean Gy (SD)**	463 (284)	431 (279)	0.16†

LBD, Largest basal tumor diameter. N/a, not available. SD, standard deviation. *Holm-Bonferroni corrected values. **Chi-square test. †Mann-Whitney *U* test. ‡Tumor center anterior to the equator, posterior to the equator, or posterior pole (within a 3 mm radius from the foveola).

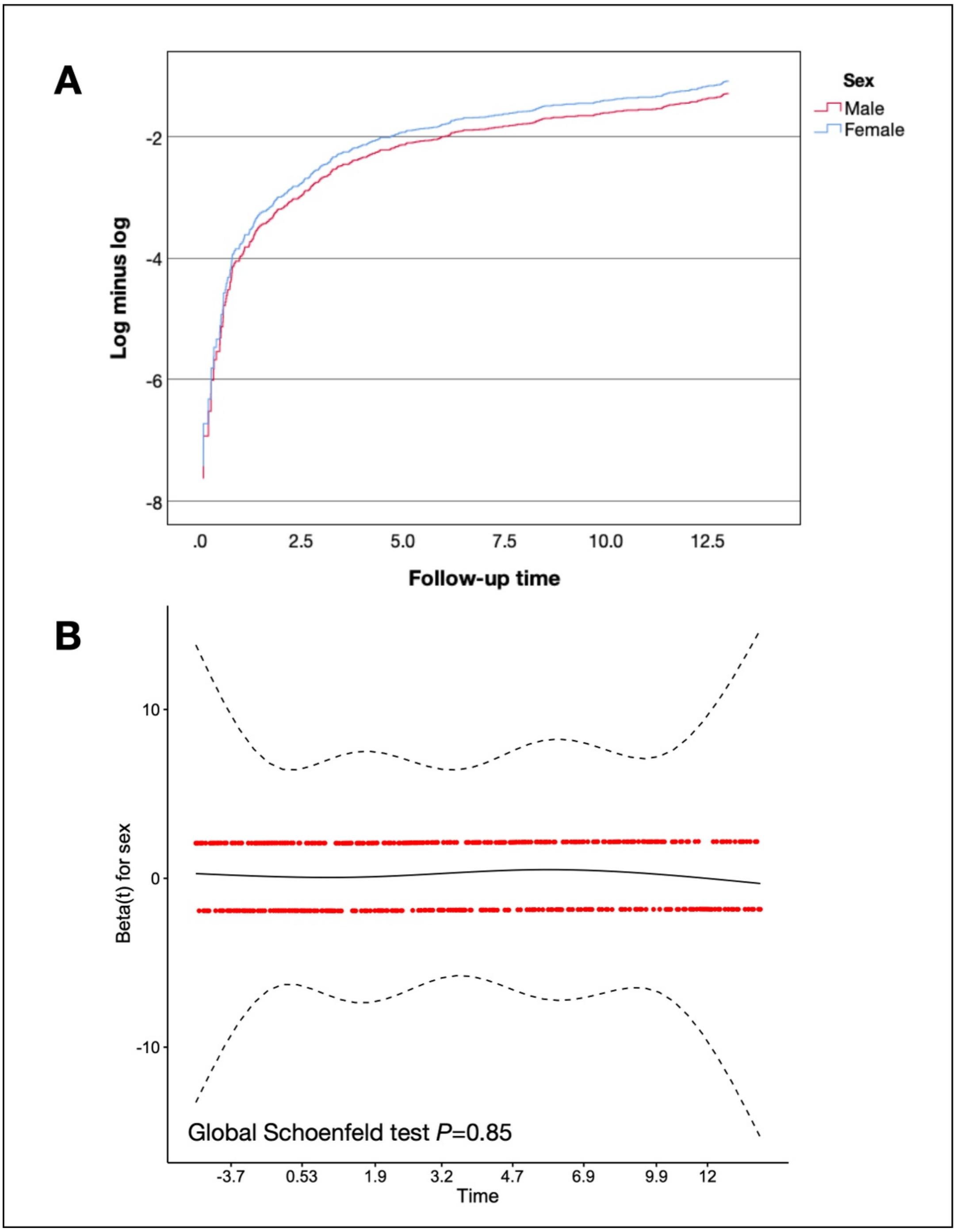

Supplementary Figure 1

Log-minus-log survival curve and Schoenfeld residuals. A) The log-minus-log survival curve for males and females was parallel without any crossing or divergence through follow-up times exceeding 1 year. B) Schoenfeld residuals plot for the delayed vs. prompt treatment covariate show no apparent trend, and the global test of proportional hazards indicated no evidence against the proportional hazards assumption.

Supplementary Table 2. Multivariate Cox regressions, hazard ratio (exp(β)) for cataract surgery.

		β_j	S.E.	*P**	exp(β)	95 % CI	Wald
**General population sample, *n*=1000**	**Multivariate**						
	Sex[a]	0.35	0.15	0.04	1.42	1.07 to 1.90	5.7
	Age[b]	0.05	0.01	<0.001	1.05	1.03 to 1.06	53.7
**Brachytherapy sample, *n*=847**	**Multivariate**						
	Sex[a]	0.08	0.15	0.63	1.08	0.80 to 1.45	0.2
	Age[b]	0.02	0.01	<0.001	1.02	1.01 to 1.04	13.9

[a]Female versus male (categorical variable). [b]Per increasing year (continuous variable). S.E., standard error. *Holm-Bonferroni corrected value.
